# Proteomic Profiling of Extracellular Vesicles Isolated From Cerebrospinal Fluid of Former National Football League Players at Risk for Chronic Traumatic Encephalopathy

**DOI:** 10.3389/fnins.2019.01059

**Published:** 2019-10-09

**Authors:** Satoshi Muraoka, Mark P. Jedrychowski, Harutsugu Tatebe, Annina M. DeLeo, Seiko Ikezu, Takahiko Tokuda, Steven P. Gygi, Robert A. Stern, Tsuneya Ikezu

**Affiliations:** ^1^Department of Pharmacology and Experimental Therapeutics, Boston University School of Medicine, Boston, MA, United States; ^2^Department of Cell Biology, Harvard Medical School, Boston, MA, United States; ^3^Department of Medical Innovation and Translational Medical Science, Kyoto Prefectural University of Medicine, Kyoto, Japan; ^4^Department of Molecular Pathobiology of Brain Diseases, Kyoto Prefectural University of Medicine, Kyoto, Japan; ^5^Department of Neurology, Alzheimer’s Disease Center, CTE Center, Boston University School of Medicine, Boston, MA, United States; ^6^Department of Neurosurgery, and Anatomy & Neurobiology, Boston University School of Medicine, Boston, MA, United States

**Keywords:** chronic traumatic encephalopathy, cerebrospinal fluid, extracellular vesicles, microtubule-associated protein tau, proteome, tauopathy, football

## Abstract

Chronic Traumatic Encephalopathy (CTE) is a tauopathy that affects individuals with a history of repetitive mild traumatic brain injury, such as American football players. Initial neuropathologic changes in CTE include perivascular deposition of phosphorylated microtubule-associated protein tau (p-tau) neurofibrillary tangles and other aggregates in neurons, astrocytes and cell processes in an irregular pattern often at the depths of the cortical sulci. In later stages, the p-tau depositions become widespread and is associated with neurodegeneration. Extracellular vesicles (EVs) are known to carry neuropathogenic molecules, most notably p-tau. We therefore examined the protein composition of EVs isolated from the cerebrospinal fluid (CSF) of former National Football League (NFL) players with cognitive and neuropsychiatric dysfunction, and an age-matched control group (CTRL) with no history of contact sports or traumatic brain injury. EVs were isolated from the CSF samples using an affinity purification kit. Total tau (t-tau) and tau phosphorylated on threonine181 (p-tau_181_) in CSF-derived EVs from former NFL players and CTRL participants were measured by ultrasensitive immunoassay. The t-tau and p-tau_181_ levels of CSF-derived EV were positively correlated with the t-tau and p-tau_181_ levels of total CSF in former NFL players, respectively, but not in the CTRL group. 429 unique proteins were identified from CSF-derived EVs and quantified by TMT-10 plex method. The identified protein molecules were significantly enriched for the extracellular exosome molecules, Alzheimer’s disease pathway and Age/Telomere Length ontology as determined by DAVID Gene Ontology analysis. Ingenuity pathway analysis of the differentially expressed EV proteins revealed enrichment of canonical liver/retinoid X receptor activation pathway. Upstream effect analysis predicted MAPT (tau) as an upstream regulator in former NFL players. These data will be useful for understanding the EV-mediated disease spread and development of novel EV biomarkers for CTE and related disorders.

## Introduction

Chronic Traumatic Encephalopathy (CTE) is a neurodegenerative tauopathy associated with repetitive mild traumatic brain injury, including concussion and subconcussion. CTE was first described in boxers in the 1920s and 1930s as “punch drunk,” (Martland, 1928) or “dementia pugilistica,” (Millspaugh, 1937) but has been more recently identified in other contact/collision sports athletes (including American football, soccer, ice hockey, and rugby players) (McKee et al., 2013, 2016; Bieniek et al., 2015; Ling et al., 2017; Mez et al., 2017; Tagge et al., 2018). Similar to other neurodegenerative diseases, CTE can only be diagnosed conclusively by neuropathologic examination. It is characterized by the perivascular deposition of phosphorylated microtubule-associated protein tau (p-tau) neurofibrillary tangles and other aggregates in neurons, astrocytes and cell processes in an irregular pattern often at the depths of the cortical sulci. In later stages, the p-tau depositions becomes widespread and is associated with neurodegeneration (McKee et al., 2015; Mez et al., 2017). CTE has a unique neuropathology from other tauopathies and the tau filaments of CTE are distinct from those in Alzheimer’s disease (AD) (McKee et al., 2016; Falcon et al., 2019).

Currently, CTE cannot be diagnosed during life. A recent study provided preliminary support for the positron emission tomography (PET) imaging p-tau ligand flortaucipir to detect CTE in living former National Football League (NFL) players (Stern et al., 2019). However, PET imaging for routine diagnostic or screening purposes is limited due to its expense and lack of availability. Accessible fluid biomarkers could be more useful for assessing the presence of CTE Cerebrospinal fluid (CSF) measures of total tau (t-tau), p-tau, and amyloid-β peptide (Ab) are widely accepted as diagnostic and prognostic biomarkers for AD (Meredith et al., 2013; Galasko and Shaw, 2017). In an initial study of CSF in the detection of CTE, there were no significant differences between former NFL players and controls in CSF t-tau and p-tau levels. However CSF t-tau in the former NFL players was associated with exposure to repetitive head impacts (RHIs) (Alosco et al., 2018).

Extracellular vesicles (EVs), including exosomes (50–150 nm), ectosomes/microvesicles (150–1000 nm), and apoptotic bodies (1000–5000 nm) are released from neurons, glia, and various other cells into the extracellular space (Perez-Gonzalez et al., 2012; DeLeo and Ikezu, 2018). They contain microRNA, mRNA, and proteins that could be transferred to recipient cells for intracellular communication. EVs are found in all bodily fluids including blood, saliva, and CSF. In the central nervous system (CNS), it has been reported that tau, Aβ, alpha-synuclein, and prion protein are presented in EVs, and that the EVs in the brain play important roles in AD, Parkinson’s disease, and prion diseases (Asai et al., 2015; Quek and Hill, 2017). In recent years, the proteins or microRNA present in EVs have emerged as an attractive target for neuronal disease detection and monitoring (Stern et al., 2016; DeLeo and Ikezu, 2018; Gill et al., 2018; Ko et al., 2018; Delpech et al., 2019). Other studies have reported proteomics analysis of EVs isolated from CSF, providing protein profiling on the composition of CSF-derived EVs as potential biomarkers for neurodegenerative diseases with high specificity (Chiasserini et al., 2014; Lee et al., 2016; Manek et al., 2018). Here, we aimed to identify a potential biomarker for diagnosing and monitoring of CTE, and we establish the proteomic profile of CSF-derived EVs in former NFL players using the ultrasensitive immunoassay and mass spectrometry.

## Materials and Methods

### Sample Selection

The CSF samples were obtained from the National Institutes of Health–funded study, “Diagnosing and Evaluating Traumatic Encephalopathy using Clinical Tests” (DETECT) at Boston University School of Medicine (BUSM). Participants included in the current study were 15 former NFL players with cognitive and neuropsychiatric symptoms and a control group (CTRL) of 16 asymptomatic same-age men without a history of contact/collision sports or traumatic brain history. The DETECT study procedures, including lumbar puncture and neuropsychological assessment, have been described elsewhere (Alosco et al., 2017, 2018). An estimate of the cumulative number of repetitive head impacts from football was calculated for NFL participants (Montenigro et al., 2017). The Institutional Review Board at Boston University Medical Campus approved the protocol and all participants provided informed consent.

### Purification of EVs From Human CSF Samples

The EV fraction was isolated from CSF samples using the MagCapture Exosome Isolation Kit PS (#293-77601 Fujifilm WAKO Pure Chemical Corporation, Japan). Briefly, the CSF samples were centrifuged at 1,200 g for 20 min at 4°C, then the supernatant was centrifuged at 10,000 g for 30 min at 4°C. The supernatant was filtered by 0.22 μm Spin-X centrifuge tube (#CLS8160 Corning, United States), then the EV fraction was isolated from the flow-through using the MagCapture Exosome Isolation Kit PS, according to the manufacturer’s instructions.

### Measurement of Total Tau (t-Tau) and Tau Phosphorylated on Threonine 181 (p-Tau_181_)

M-PER^®^ Mammalian Protein Extraction Reagent (#78503 PIERCE) was added to the isolated EV fraction using MagCapture Exosome Isolation Kit PS with Halt^TM^ Protease and Phosphatase Inhibitor Cocktail (#78442 Thermo Fisher Scientific, United States) and was mixed by vortexing for 15 min. The lysed EVs were filtered by 0.45 μm Spin-X centrifuge tube (#CLS8162 Corning, United States). The EV t-tau and p-tau_181_ were measured using modified the Human Total Tau Simoa kit (Quanterix, Lexington, MA) on the Simoa HD-1 analyzer (Quanterix). Briefly, this kit is an updated version of the Simoa assay. It uses a monoclonal capture antibody that reacts with an epitope in the mid-region of all tau isoforms in combination with a detection antibody that reacts with an epitope at the N-terminus of t-tau for t-tau immunoassay or in PHF-tau (AT270, Thermo Fisher Scientific) for p-tau_181_ immunoassay. We used t-tau or human tau (p-tau_181_) as standards in the Human Tau ELISA^TM^ kit or Human Tau [pT181] phosphoELISA^TM^ ELISA kit (Invitrogen, Thermo Fisher Scientific) for each assay, respectively (Tatebe et al., 2017). All CSF-derived EV samples were diluted 4x with the Tau 2.0 Sample Diluent (Invitrogen, Thermo Fisher Scientific) prior to the assays, to minimize matrix effects, and were analyzed in duplicate on one occasion. The relative concentration estimates of t-tau and p-tau_181_ were calculated according to the standard curve. For CSF t-tau and p-tau_181_, the levels were measured using the multiplex xMAP Luminex platform (Luminex Corp., Austin, TX, United States) with Fujirebio (INNO-BIA AlzBio3, Ghent, Belgium) immunoassay kit-based reagents (Alosco et al., 2018).

### Nanosight Tracking Analysis (NTA)

All samples were diluted in double-filtered PBS (dfPBS, with 0.22 μm pore-size) at least 1:10 to obtain particles within the target reading range for the Nanosight 300 machine (Malvern Panalytical Inc.), which was 10-100 particles per frame. Using a stage pump system four 30-s videos were taken for each sample at 21°C. Analysis of particle counts was carried out in the Nanosight NTA 3.2 software (Malvern Panalytical Inc.) with a detection threshold of 5.

### SDS-PAGE and In-Gel Digestion

Ice-cold 100% (w/v) trichloroacetic acid (TCA) (#T6399 Sigma-Aldrich) was added to the isolated EV fraction to a final concentration of 20% of TCA, then the mixed sample was incubated for 30 min on ice and was centrifuged at 15,000 g for 20 min at 4°C. The pellet was then washed twice with ice-cold acetone (#179124 Sigma-Aldrich). After drying, the pellet was resuspended in Laemmli sample buffer (#1610747 Bio-Rad) with 5 mM dithiothreitol (# 43815 Sigma-Aldrich), reduced for 20 min at 65°C, and alkylated with 15 mM iodoacetamide (# I1149 Sigma-Aldrich) for 20 min at room temperature in the dark. Subsequently, the samples were run in a 4–20% gradient gel (#4561096 Bio-Rad) until the dye front was 10 mm from the top of the gel. The gels were washed twice with distilled water, fixed with 100% Methanol, and stained with GelCode Blue Stain Reagent (#24590 Thermo Fisher Scientific) for 16 hrs. Each lane was then individually removed from the gel. Gel pieces were then transferred to 1.5 mL tubes and destained twice using 50% acetonitrile (J. T. Baker, United States) in 25 mM HEPES (pH 8.8) at 22°C, for 15 min with shaking, and dehydrated with 100% acetonitrile for additional 10 min with shaking, for a total of three times. The destained gel piece was dried up using SpeedVac Concentrators (Thermo Fisher Scientific). The gel pieces were digested with proteomic grade trypsin (#03708985 Roche, United States) in 25 mM HEPES overnight at 37°C. The digested peptide was extracted with 70% acetonitrile/1% formic acid, and were removed the gel by Ultrafree-MC Centrifugal Filter (#UFC30L Millipore United States). The digested peptides were reconstituted in 25 μL of 200 mM EPPS (pH 8.0) and vortexed for 5 min.

### Peptide Labeling With TMT 10-Plex Isobaric Labeling Kit

Tandem mass tag (TMT) labeling was performed according to the manufacturer’s instructions (#90409 Thermo Fisher Scientific). In brief, 4 μL of TMT reagent (20 ng/μL) was added to the digested peptides. Following incubation at room temperature for 1 h, the reaction was quenched with 2 μL of 5% hydroxylamine for 15 min. The TMT-labeled peptide samples were pooled at a 1:1 ratio across 10 samples. The combined sample was added into 100 μL of 20% formic acid, 2 mL of 1% formic acid, desalted via StageTip, dried by vacuum centrifugation, and resuspended in 5% acetonitrile and 5% formic acid for nano-liquid chromatography and tandem mass-spectrometry (NanoLC-MS/MS).

### Nano-Liquid Chromatography and Tandem Mass-Spectrometry

NanoLC–MS/MS analysis was conducted by an LTQ-Orbitrap Fusion Lumos mass spectrometer (Thermo Fisher Scientific) equipped with a Proxeon EASY-nanoLC 1200 liquid chromatography pump (Thermo Fisher Scientific, San Jose, CA, United States). Peptides were separated on a 100 μm inner diameter microcapillary column packed with ∼40 cm of Accucore 150 resin (2.6 μm, 150 Å, Thermo Fisher Scientific). We loaded 4 μL onto the column and separation was achieved using a 180 min gradient of 8 to 23% acetonitrile in 0.125% formic acid at a flow rate of ∼550 nL/min. The analysis used an MS3 based TMT method, which has been shown to reduce ion interference. The scan sequence began with an MS1 spectrum (Orbitrap; resolution 120,000; mass range 400–1400 m/z; automatic gain control (AGC) target 5 × 10^5^; maximum injection time 100 ms). Precursors for MS^2^/MS^3^ analysis were selected using a Top10 method. MS2 analysis consisted of collision-induced dissociation (quadrupole ion trap; AGC 2 × 10^4^; normalized collision energy (NCE) 35; maximum injection time 150 ms). Following acquisition of each MS^2^ spectrum, we collected an MS^3^ spectrum using our recently described method in which multiple MS^2^ fragment ions were captured in the MS^3^ precursor population using isolation waveforms with multiple frequency notches. MS^3^ precursors were fragmented by high energy collision-induced dissociation (HCD) and analyzed using the Orbitrap (NCE 65; AGC 1 × 10^5^; maximum injection time 150 ms, resolution was 50,000 at 200 Th).

### Mass-Spectrometry Data Analysis

A compendium of in-house developed software was used to convert mass spectrometric data (Raw file) to the mzXML format, as well as to correct monoisotopic m/z measurements (Elias and Gygi, 2007). Database searching included all entries from the *Homo sapiens* UniProt database (downloaded October, 2018). This database was concatenated with one composed of all protein sequences in the reversed order. Searches were performed using a 50 ppm precursor ion tolerance for total protein level profiling (McAlister et al., 2014). The product ion tolerance was set to 0.9 Da. These wide mass tolerance windows were chosen to maximize sensitivity in conjunction with SEQUEST searches and linear discriminant analysis. TMT tags on lysine residues and peptide N termini (+ 229.163 Da) and carbamidomethylation of cysteine residues (+ 57.021 Da) were set as static modifications, while oxidation of methionine residues (+ 15.995 Da) was set as a variable modification. Peptide-spectrum matches (PSMs) were adjusted to a 1% false discovery rate (FDR). Filtering was performed using an in-house linear discrimination analysis (LDA) method to create one combined filter parameter from the following peptide ion and MS2 spectra metrics: SEQUEST parameters XCorr and ΔCn, peptide ion mass accuracy and charge state, in-solution charge of peptide, peptide length and mis-cleavages. Linear discrimination scores were used to assign probabilities to each MS2 spectrum for being assigned correctly and these probabilities were further used to filter the dataset with an MS2 spectra assignment FDR of smaller than a 1% at the protein level (Huttlin et al., 2010). For TMT-based reporter ion quantitation, we extracted the summed signal-to-noise (S/N) ratio for each TMT channel and found the closest matching centroid to the expected mass of the TMT reporter ion. PSMs were identified, quantified, and collapsed to a 1% peptide false discovery rate (FDR) and then collapsed further to a final protein-level FDR of 1%. Moreover, protein assembly was guided by principles of parsimony to produce the smallest set of proteins necessary to account for all observed peptides. Proteins were quantified by summing reporter ion counts across all matching PSMs. PSMs with poor quality, MS^3^ spectra with more than eight TMT reporter ion channels missing, MS^3^ spectra with TMT reporter summed signal-to-noise ratio less than 100, or no MS^3^ spectra were excluded from quantification. The mass spectrometry proteomics data have been deposited to the ProteomeXchange Consortium via the PRIDE partner repository with the dataset identifier PXD015358 (Perez-Riverol et al., 2019). Protein quantitation values were exported for further analysis in Microsoft Excel or Prism 6. Each reporter ion channel was summed across all quantified proteins.

### Statistical Analysis

Statistical analysis was conducted using IBM SPSS software ver.25 and GraphPad Prism 6. Between group comparisons were analyzed by non-parametric Mann-Whitney U or one-way ANOVA followed by Bonferroni correction for multiple comparisons. Bivariate correlation analysis examined differences between former NFL players and controls in EV t-tau, EV p-tau_181_, proteomics data, and demographics data using IBM SPSS software ver.25. The Gene Ontology of identified proteins were elucidated by DAVID Bioinformatics Resources 6.8. The Protein networks and pathway analysis were generated using Ingenuity Pathway Analysis (IPA)^1^. The Venn diagram and Heatmap analysis were generated using Venny_2.1^2^ and MeV_4_8^3^.

## Results

### Workflow for Proteome Analysis of Former NFL Players CSF Derived EVs

**FIGURE 1:**
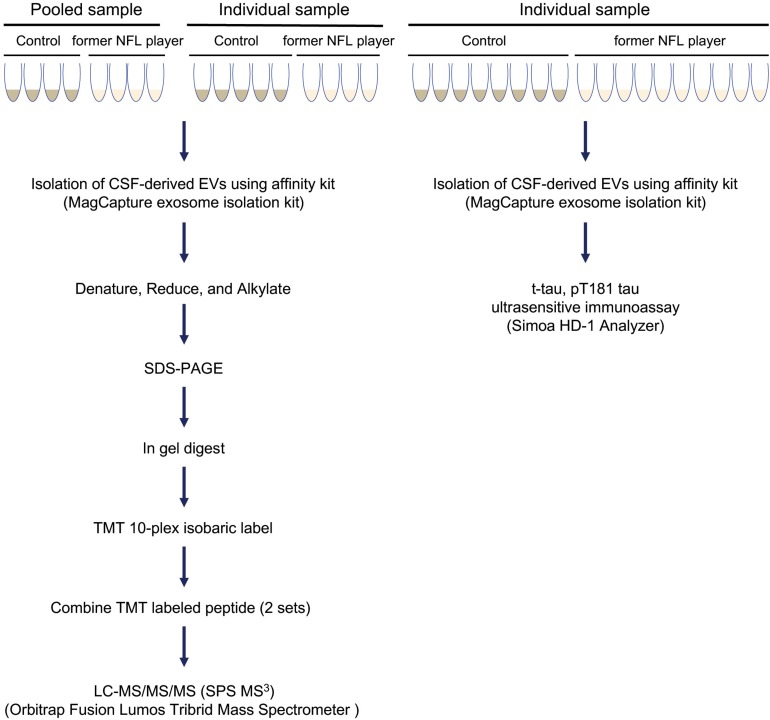
Workflow used in proteomics analysis of former NFL players CSF-derived EVs: EVs were isolated from control and former NFL players CSF using MagCapture Isolation kit. For pooled proteomics analysis, 4 pooled samples from former NFL players or CTRLs were tested. For individual proteomics analysis, samples from 4 former NFL players and 5 CTRLs were tested. The isolated EVs were denatured, reduced, and alkylated and run on 4–20% gradient SDS-PAGE gel. The protein band was cut out of the gel followed by trypsin digestion and labeled with TMT 10-plex isobaric label kit. The combing TMT-labeled peptide was analyzed by MS^3^ on Orbitrap Fusion Lumos Tribrid Mass Spectrometer. For immunoassays, EV samples from 10 former NFL players and 8 CTRLs were tested. Total tau (t-tau) and tau phosphorylated at threonine 181 (p-tau_181_) in CSF-derived EV or total CSF samples were measured by ultrasensitive or conventional immunoassay (Simoa or multiplex Luminex systems), respectively.

The experimental workflow is summarized in Figure 1. The EVs were isolated from former NFL players CSF and an age-matched control group (CTRL) with no history of contact sports or traumatic brain injury using the MagCapture Exosome isolation kit. For EV t-tau and p-tau_181_ immunoassays, the 10 former NFL players and 8 CTRLs isolated EVs were lysed with M-Per lysis buffer and then measured the levels of t-tau and p-tau_181_ in EVs or total CSF were measured by ultrasensitive or conventional immunoassay (see Materials and Methods) (Supplementary Table S1). For the proteome profiling, the CSF samples were analyzed as (1) 4 pooled samples from either 2 or 4 former NFL players or CTRL individuals and as (2) individual samples from 4 former NFL players and 5 CTRLs (Table 1 and Supplementary Table S1). The isolated EVs were run in the SDS-PAGE for in-gel digestion, the digested peptides were then labeled with TMT 10-plex isobaric labeling kit, and analyzed by high sensitivity mass spectrometry (see Materials and Methods).

**TABLE 1:** Patient information.

	**Control (*n* = 16)**	**NFL player (*n* = ’15)**	***p*-value^a^**
Age, mean	57.06 ± 6.95	56.33 ± 7.31	0.7178
BMI, mean	27.81 ± 3.43	33.07 ± 4.66	0.0008
Duration of football play, mean year	–	18.87 ± 3.96	–
Years in NFL, mean year	–	8.40 ± 3.48	–
Cumulative Head Impact Index^b^	–	20728.46 ± 6739.56	–

*^*a*^The statistical significance of the differences were calculated using Mann-Whitney test. ^*b*^Estimated number of football-related repetitive head impacts (Montenigro et al., 2017).*

### Biochemical Characterization of Former NFL Players CSF-Derived EVs

**FIGURE 2:**
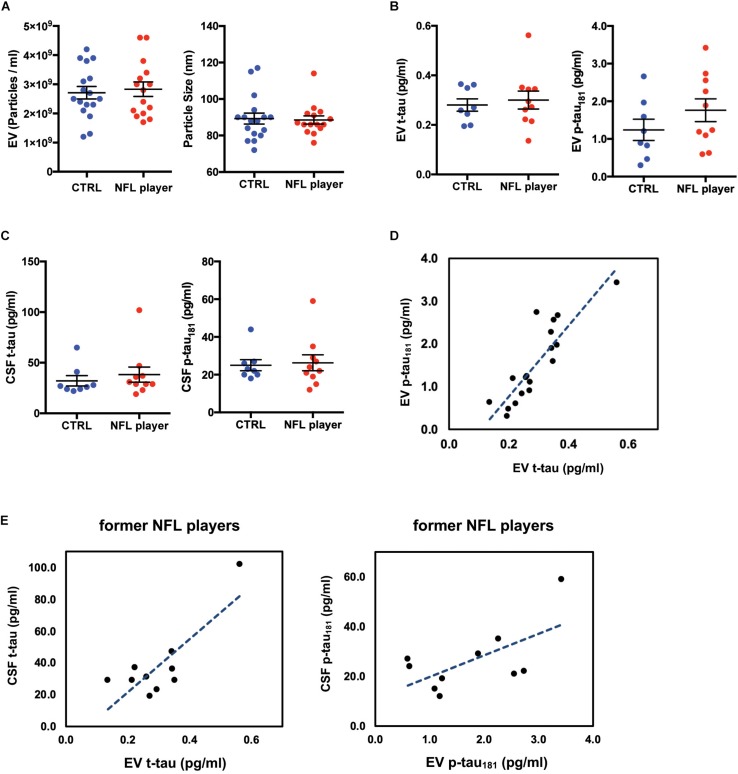
Biochemical characteristic of CSF-derived EVs isolated from former NFL players. **(A)** Left: Particle numbers of CSF-derived EV fraction from CTRL or former NFL players by Nanoparticle tracking analysis (*p* = 0.9628 by Mann-Whitney test). Right: Particle size of CSF-derived EV fraction (*p* = 0.9628). **(B)** T-tau and p-tau_181_ levels in CSF-derived EVs by ultrasensitive immunoassay. Left: EV t-tau levels (*p* = 0.8688). Right: EV p-tau_181_ levels (*p* = 0.2682). **(C)** T-tau and p-tau_181_ levels in total CSF by multiplex Luminex platform. Left: CSF t-tau (*p* = 0.3031). Right: CSF p-tau_181_ (*p* = 0.9477). **(D)** Scattered plot of t-tau and p-tau_181_ in CSF derived EV samples (*r* = 0.870, *p* < 0.0001). **(E)** Left: Scattered plot of t-tau levels in CSF-derived EV and in total CSF in the former NFL players (*r* = 0.812, *p* = 0.0044). Right: Scattered plot of p-tau_181_ levels in CSF-derived EV and in total CSF in the former NFL players (*r* = 0.627, *p* = 0.0524).

To measure the concentration and size of EVs in CSF, we examined former NFL players and control CSF-derived EVs using nanoparticle tracking analysis (NTA, Supplementary Figure S1). The EV concentrations in former NFL players and CTRLs CSF had no significant difference (*p* = 0.9628). The mode size distribution peaked at 86 nm in former NFL players CSF and at 88 nm in CTRLs (*p* = 0.9628) (Figure 2A). To characterize the CSF-derived EVs, we isolated EVs from the CSF samples of former NFL players and CTRLs using the MagCapture Exosome Isolation kit. First, we measured the concentration of total tau (t-tau) and phosphorylated tau protein at threonine 181 (p-tau_181_) in isolated EVs and total CSF from the 10 former NFL players and 8 CTRLs by ultrasensitive immunoassay or multiplex Luminex assay. Both t-tau and p-tau could be detected in both former NFL players and CTRLs CSF-derived EVs and total CSF samples. However, there was no significant difference between former NFL players and CTRLs (t-tau: *p* = 0.8688 and p-tau_181_: *p* = 0.2682 in CSF-derived EVs; t-tau: *p* = 0.3031 and p-tau_181_: *p* = 0.9477 in total CSF) (Figures 2B,C). The level of EV p-tau_181_ was significantly correlated with EV t-tau (*r* = 0.870, *p* < 0.0001) (Figure 2D). Unexpectedly, the EV t-tau levels were lower than those of EV p-tau_181_. One possible explanation is that the majority of tau in the CSF EVs is fragmented. Next, we assessed the correlation of CSF-derived EV t-tau, p-tau_181_ and total CSF t-tau, p-tau_181_ by Pearson’s correlation analysis. There is a positive correlation between CSF t-tau, p-tau_181_ and EV t-tau, p-tau_181_ levels in former NFL players (t-tau: *r* = 0.812, *p* = 0.0044; p-tau_181_: *r* = 0.627, *p* = 0.0524) (Figure 2E), but not in the CTRL group (t-tau: *r* = −0.492, *p* = 0.2150; p-tau_181_: *r* = −0.530, *p* = 0.1770) (data not shown).

### TMT-Based Proteomic Analysis of EV Proteins Isolated From the CSF of Former NFL Players and CTRL Group

**FIGURE 3:**
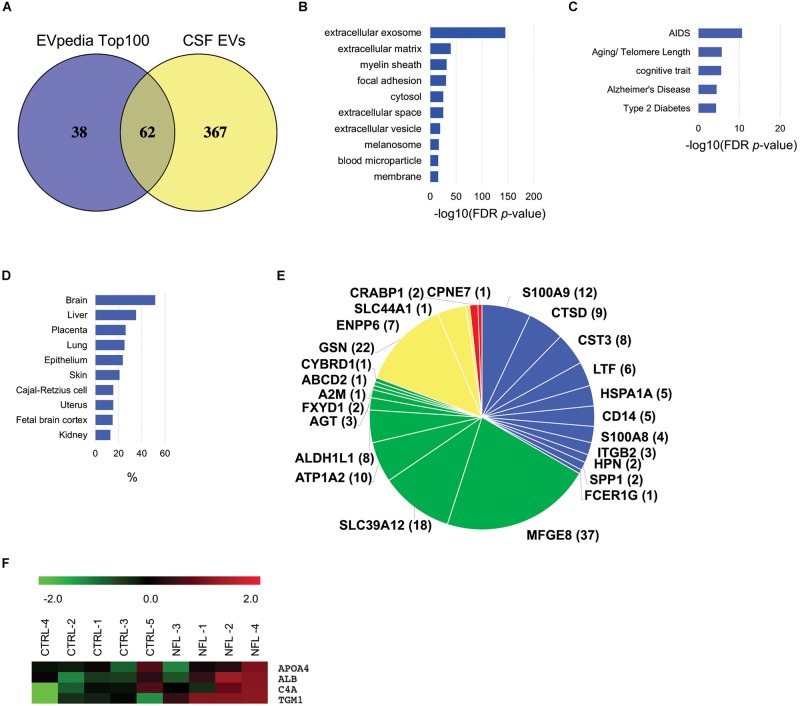
Bioinformatic characterization of former NFL players CSF-derived EV proteome dataset: **(A)** Venn diagram of the 429 proteins identified in CSF-derived EV and EVpedia Top 100. **(B)** Gene Ontology (GO) analysis using DAVID Bioinformatics Resources 6.8. The GO term of Top10 Cellular Component with –log10 (FDR *p*-value). **(C)** The GO term of Top5 Disease Ontology with –log10 (FDR *p*-value). **(D)** The GO term of Top10 Tissue Expression Ontology with% enrichment. **(E)** Enrichment of brain cell-specific markers in CSF-derived EV proteins. The brain cell-specific markers were searched using Barres Brain RNA-Seq datasets. Blue: Microglia, Green: Astrocytes, Yellow: Oligodendrocytes, Red: neurons. The parentheses show the number of identified peptides by LC-MS/MS. **(F)** Heatmap of EV proteins in individual proteome dataset. Each row in the heat map show a protein, and each column represent CTRL and former NFL players. Red indicate up-regulate expression and Green show down-regulate.

To generate EV protein profiling of former NFL players’ CSF, we analyzed EV proteins isolated from the former NFL players and CTRL cohorts by NanoLC-MS/MS using TMT-based labeling (Paulo et al., 2018). We identified a total of 429 unique proteins across both cohorts (Supplementary Tables S2, S3). The identified proteins were compared with the top 100 EV proteins from the EVpedia database (Kim et al., 2015). The comparison revealed that 62 of the top 100 EV proteins were expressed in the CSF-derived EVs (Figure 3A). The 381 proteins remaining after exclusion of keratins and immunoglobulins were examined with respect to Cellular component, Tissue expression, and Disease annotation by Gene Ontology analysis in the Database for Annotation, Visualization and Integrated Discovery (DAVID). Among these 381 proteins, 73.9% of them were annotated to be the Extracellular exosome ontology (Figure 3B), indicative of the purity of our CSF-derived EV samples. Furthermore, 11.8% of the identified molecules were enriched for the Alzheimer’s disease or Aging/Telomere Length pathway, which was related to AD and senescence-associate genetic alteration (Figure 3C). The EV samples were enriched in exosomal markers, including Rab GTPases, immune receptors, flotillins, annexins, tetraspanins, apolipoproteins, heat shock proteins, and ESCRT associated proteins (Table 2). Using tissue expression analysis, we observed enrichment of 196 proteins (51.6%) derived from brain tissues (Figure 3D). We searched for brain cell-specific markers within the EV proteomics dataset using the Brain RNA-Seq database created by Barres Lab (Zhang Y. et al., 2014). Several proteins in our dataset were identified in the database as enriched markers for microglia (S100A9, CTSD, CST3, LTF, HSPA1A, CD14, S100A8, ITGB2, HPN, SPP1, FCER1G), astrocyte markers (MFGE8, SLC39A12, ATP1A2, ALDH1L1, AGT, FXYD1, A2M, ABCD2, CYBRD1), oligodendrocyte markers (GSN, ENPP6, SLC44A1) and neuron markers (CRABP1, CPNE7) (Figure 3E). These data indicated that the CSF-derived EVs were secreted from brain cells, and presumably mainly from glial cells. Using individual proteome dataset, we investigated differences in the expression of EV proteins between the 4 former NFL players and 5 CTRL groups. However, no molecules were significantly altered between former NFL players and control groups based on the criteria of at least >1.5 or <0.67-fold-change with an adjusted *p* value of <0.05. Next, we performed semi-supervised hierarchical clustering of differentially expressed proteins across the individual proteomics dataset using Pearson Correlation metric and Average Linkage clustering as parameters for the Raw (proteins). Proteins cluster if they are differentially expressed between former NFL players and CTRLs (Figure 3F). The TGM1 protein, which was colocalized with aggregated Ab in senile plaques (SPs) and tau in Neurofibrillary tangles (NFTs) (Wilhelmus et al., 2009), in the cluster showed significant difference in expression level, but did not meet the pre-specified fold change cut-off (fold change = 1.18, *p* = 0.0259).

**TABLE 2:** Identification of exosomal markers in CSF-derived EV.

		**Pooled proteome**	**Individual proteome**
			
**Family^a^**	**Gene name**	**Identified peptide^b^**	**Identified peptide**
RAB	RAB10	1	0
IR	HLA-A	4	1
	HLA-DRA	3	2
	HLA-DRB1	2	2
	HLA-DRB5	2	0
	CD14	3	2
	CD59	7	4
FLOT	FLOT1	1	0
ANXA	ANXA1	3	5
	ANXA2	26	14
	ANXA4	3	4
	ANXA5	12	12
	ANXA6	11	5
	ANXA7	3	0
	ANXA11	3	1
TSPAN	CD9	9	3
	CD81	8	3
	CD82	2	0
	TSPAN4	0	2
APO	APOA1	18	1
	APOA4	6	7
	APOD	11	1
	APOE	26	18
HSP	HSPA1A	4	1
	HSPA8	18	11
	HSPB1	3	3
	HSPD1	8	0
	HSPE1	2	0
	HSP90AA1	10	1
	HSP90AB1	7	0
ESCRT-AP	PDCD6IP	18	13
	SDCBP	16	9

*^*a*^RAB, Rab GTPases; IR, immune receptor; FLOT, flotillin; ANXA, annexin; TSPAN HSP, tetraspanin; APO, apolipoprotein; HSP, heat shock prottein; ESCRT-AP, ESCRT associated proteine. ^*b*^The identified peptid shows count by LC-MS/MS.*

### Networks Analysis for Former NFL Players CSF-Derived EV Individual Proteome Dataset

**FIGURE 4:**
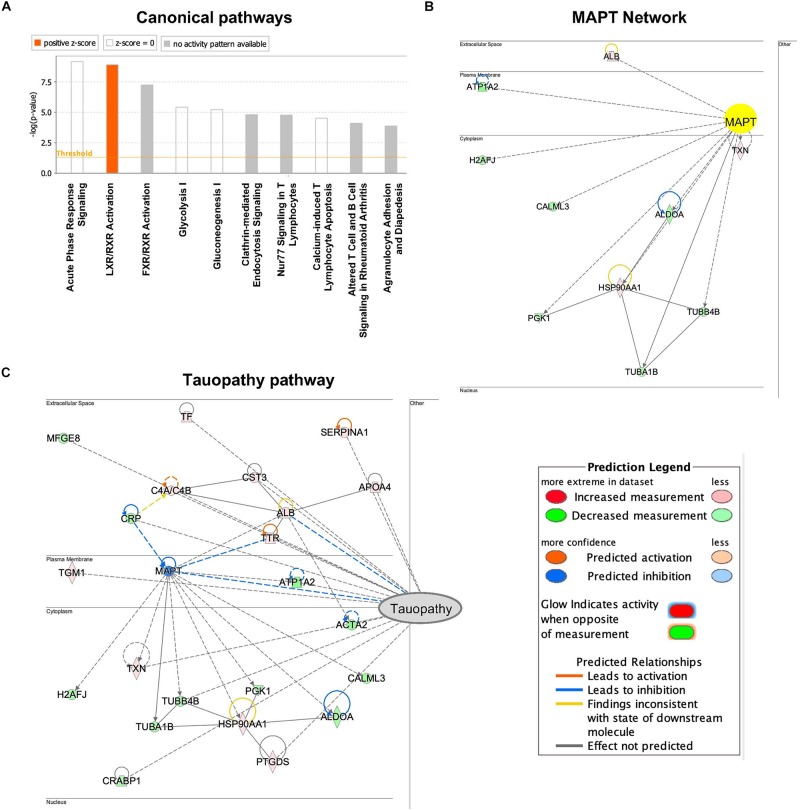
Networks produced by Ingenuity pathway analysis based on CSF-derived EV proteome dataset from individual former NFL players: **(A)** The canonical pathways that were up- and down-regulated in former NFL players compared to controls. Upregulated pathways are denoted in red. **(B)** Upstream effect analysis of differentially expressed EV proteins in former NFL players. IPA predicted MAPT (tau) as activated and suppressed upstream regulator protein. Up-regulated protein is denoted in red and down-regulated protein is shown green. Solid and dashed lines are indicated direct and indirect interactions, respectively. **(C)** IPA-based protein network in neurological disease based on tauopathy. The intensity of the node color shows the degree of up–regulation (red) or down–regulation (green).

To assess enriched canonical pathways, upstream, and functional networks in the differentially expressed 61 EV proteins in the former NFL players (fold-change: >1.2 or <0.83), we analyzed the individual sample datasets using Ingenuity Pathway Analysis (IPA). Among top 10 differentially regulated canonical pathway, liver/retinoid X receptor (LXR/RXR) activation pathway, which was associated with ALB, C4A, FGA, S100A8, TF, TTR and SERPINA1, was upregulated in the former NFL players (Figure 4A and Table 3). Interestingly, MAPT (tau) was predicted as an upstream regulator protein for 10 differentially expressed EV proteins in the former NFL players (Figure 4B). Finally, we identified the signaling network for the connecting the tauopathy and MAPT regulating proteins (Figure 4C), suggesting the potential use of these molecules for the assessment of molecular changes induced by tau accumulation or tauopathy development in the CNS.

**TABLE 3:** Canonical pathways generated from protein lists comprised of significant proteins by Ingenuity Pathway Analysis.

**Canonical pathway**	**Associated with the canonical pathway^a^**	**Ratio**	**−log (*p*-value)**
Acute phase response signaling	ALB, C4A, CRABP1, CRP, FGA, FN1, SERPINA1.TF, TTR	9/178	9.178
LXR/RXR activation	ALB, APOA4, C4A, FGA, S100A8, SERPINA1, TF, TTR	8/128	8.928
FXR/RXR activation	ALB, APOA4, C4A, FGA, SERPINA1, TF, TTR	7/137	7.256
Glycolysis 1	ALDOA, C3, PGK1, C9	4/42	5.412
Gluconeogenesis 1	ALDOA, C3, PGK1, C9	4/47	5.213
Clathrin mediated endocytosis signaling	ACTA2, ALB, APOA4, S100A8, SERPINA1, TF	6/212	4.796
77 signaling in T lymphocytes	CALML5, FCER1G, HLA-A, HLA-DRB1	4/60	4.788
Calcium-induced T lymphocyte apoptosis	CALML5, FCER1G, HLA-A, HLA-DRB1	4/70	4.521
Altered T cell and B cell signaling in rheumatoid arthritis	FCER1G, HLA-A, HLA-DRB1, SPP1	4/90	4.093
Agranulocyte adhesion and diapedesis	ACTA2, FN1, GNAI2, MYH9, RDX	5/193	3.886

*^*a*^ALB, albumin; C4A, complement C4-A; CRABP1, cellular retinoic acid-binding protein 1; CRP, C-reactive protein; FGA, fibrinogen alpha chain; FN1, fibronectin; SERPINA1, alpha-1-antitrypsin; TF, Serotransferrin; TTR, transthyretin; APOA4, apolipoprotein A-IV; S100A8, Protein S100-A8; ALDOA, fructose-bisphosphate aldolase A; C3, complement C3; PGK1, phosphoglycerate kinase 1; C9, complement C9; ACTA2, actin aortic smooth muscle; FCER1G, high affinity immunoglobulin epsilon receptor subunit gamma; HLA-A, HLA class I histocompatibility antigen A-2 alpha chain; HLA-DRB1, HLA class II histocompatibility antigen DRB1-14 beta chain; SPP1, osteopontin; GNAI2, guanine nucleotide-binding protein G(i) subunit alpha-2; MYH9, myosin-9; RDX, radixin.*

## Discussion

In this study, we performed digital ELISA analysis of t-tau and p-tau_181_ and NanoLC–MS/MS analysis in CSF-derived EVs from former NFL players for the first time. We found that t-tau and p-tau_181_ levels of CSF-derived EV were positively correlated with the t-tau and p-tau_181_ levels of total CSF in former NFL players, but not in the CTRL group. We also identified 429 unique proteins from CSF-derived EVs, which were significantly enriched for the extracellular exosome molecules, Alzheimer’s disease pathway and Age/Telomere Length ontology as determined by DAVID Gene Ontology analysis. Ingenuity pathway analysis of the differentially expressed EV proteins revealed enrichment of canonical liver/retinoid X receptor activation pathway. Finally, upstream effect analysis predicted MAPT (tau) as an upstream regulator in former NFL players.

As for tau levels in the CSF-derived EVs, the t-tau levels in the CSF-derived EV samples was lower than expected and accounted for less than 20% of p-tau_181_ levels. One explanation for this finding is that EVs in CSF might contain an N-terminal-deleted fragment of tau, which might be undetected by the tau ELISA kit. We used a commercial Human Tau ELISA^TM^ kit and modified Human Tau [pT181] phosphoELISA^TM^ kit for this study. The total tau ELISA kit uses a capture antibody that reacts with mid-region of tau and a detection antibody that recognizes the N-terminal region. However, the modified phosphoELISA kit uses a capture antibody that recognizes the mid-region of tau and a detection antibody that reacts with p-tau_181_. When t-tau and p-tau_181_ in the CSF from the same former NFL players and CTRLs were measured by Fujirebio kit, which uses mid-region capture antibody for t-tau or p-tau_181_ specific antibody in mid-region for p-tau and mid-region-specific detector antibody for both t-tau and p-tau detection, the CSF level of t-tau was higher than p-tau. Chen et al. (2019) reported that N-terminal and mid-region containing fragments of tau were elevated in CSF with AD and MCI, but did not detect full-length tau in the CSF. Other studies indicated that the N-terminal fragments of tau were secreted by activated neurons, which was increased in CSF from AD patients (Chen et al., 2019; Cicognola et al., 2019). Moreover, Zhao et al. (2016) reported that caspase-2-cleaved tau fragment were elevated in AD tissue, which was infiltrated into dendritic spines and ultimately induced memory dysfunction in their mouse models. On other hands, in the hippocampal tissue of AD patients, Calpain 1 and 2-cleaved tau fragments were found, but could not induce their phosphorylation or cytotoxicity (Ferreira and Bigio, 2011; Garg et al., 2011). In addition to caspase and calpain, thrombin, asparagine endopeptidase and cathepsins have been reported to be tau-cleaving enzymes (Zhang Z. et al., 2014). Calpain-1 and Caspase-14 were identified in CSF-derived EVs in the present proteome analysis; therefore, this suggests that CSF-derived EVs might be contained N-terminal-deletion fragments of tau. Future studies are necessary to determine whether fragments of tau are present in specific brain cell type-specific derived EVs within CSF.

There is accumulating interest in proteomic analysis of CSF and CSF-derived EVs to identify biomarkers for neurodegenerative disorders. Here, we report the first proteomic analysis of CSF-derived EV samples isolated from former NFL players. Our proteomic analysis reports a slightly higher enrichment rate of EV-specific molecules in our CSF-derived EV preparation than previous reports, despite the lower number of identified molecules (Street et al., 2012; Chiasserini et al., 2014; Lee et al., 2016; Manek et al., 2018). The MagCapture exosome isolation kit employed here has been reported to have a higher purity of EV than other exosome isolation kits, resulting in the lower protein yields (Nakai et al., 2016). For these reasons, although we used 3 mL of CSF per sample in the present study, the number of identified proteins was lower than other have reported. In this individual proteomics study, we analyzed 4 former NFL players and 5 CTRL groups but found no significant differences between the groups. This could be due to the small sample size and some of the NFL players may not have CTE although they are high risk group. A further study is necessary with increased sample size and follow up with the former NFL players. When our proteomics dataset was compared with the CSF proteome dataset, only 114 proteins were commonly detected between total CSF and CSF-derived EVs, and they were mostly non-EV molecules (Dayon et al., 2019). On the other hand, our proteomic profiling data indicated that CSF-derived EVs are enriched in molecules from microglia, astrocytes, oligodendrocytes, and neurons to a lesser extent. Moreover, in CSF derived EVs, proteins specific to such brain cell as microglia (S100A9, CTSD, CST3, LTF, HSPA1A, CD14, S1008, ITGB2, SPP1), astrocytes (MFGE8, SLC39A12, ATP1A2, AGT, A2M), oligodendrocytes (GSN), and neurons (CRABP1) were identified commonly in the present study as well as by previously published proteomics studies on pooled human CSF samples (Chiasserini et al., 2014). Canonical pathway analysis revealed that enrichment of acute phase response and LXR/RXR activation, suggesting the elevation of inflammatory conditions and lipid biosynthesis pathway in the CSF-derived EVs from former NFL players. Researchers have reported the LXR/RXR pathway has important neuroprotective roles in neurodegenerative disease, including stroke, ALS, AD, and PD (Nam et al., 2016). In AD, LXR/RXR pathway has a role in microglial activity or in clearance of amyloid beta from the brain (Moutinho and Landreth, 2017). In former NFL players with risk of CTE, the LXR/RXR pathway might be able to affect neuronal survival and regulate microglia activity. Future exploration is needed to elucidate the activation of this pathway in CTE.

In summary, we have quantified tau levels and proteomic profiles in CSF-derived EVs from former NFL players in this study, identified unique enrichment of Alzheimer’s disease pathway and Age/Telomere Length ontology, canonical liver/retinoid X receptor activation pathway, and predicted MAPT (tau) as an upstream regulator in former NFL players. Fluid and neuroimaging biomarkers have been mainly focused on tau: Tau PET imaging has been developed as an important tool for CTE diagnosis (Stern et al., 2019) and total tau and exosomal tau were identified as blood-based biomarker in CTE (Stern et al., 2016). The CSF is an attractive biofluid for biomarker discovery research with abundant proteins associated with neurodegenerative diseases in early stages in comparison to blood samples (Alosco et al., 2018). Our results suggested that p-tau_181_ in CSF-derived EVs and a combination of several cell type-specific molecules are potential monitoring biomarkers in former NFL players at risk for CTE. Further investigations are necessary to confirm these biomarkers, including additional validation sets, and to clarify the implications of the correlation of this marker with disease, and to elucidate the mechanisms of EV secretion from the brain cell types in former NFL players.

## Data Availability Statement

The datasets generated for this study can be found in the ProteomeXchange Consortium via the PRIDE partner repository with the dataset identifier PXD015358 (http://www.proteomexchange.org).

## Ethics Statement

The studies involving human participants were reviewed and approved by the Boston University School of Medicine. The patients/participants provided their written informed consent to participate in this study.

## Author Contributions

SM, RS, and TI designed the research. SM, MJ, HT, and AD performed the research. SM, MJ, SG, TT, and TI analyzed the data. RS provided the CSF samples. SM and TI wrote the manuscript. SM, MJ, AD, SI, RS, and TI edited the manuscript.

## Conflict of Interest

TI has sponsored research agreement from Aethlon Medical, Inc., for this study. The remaining authors declare that the research was conducted in the absence of any commercial or financial relationships that could be construed as a potential conflict of interest.

## Footnotes

1 https://www.qiagenbioinformatics.com/products/ingenuity-pathway-analysis/

2 http://bioinfogp.cnb.csic.es/tools/venny/

3 http://mev.tm4.org

## Supplementary Material

FIGURE S1 NTA plot of average size and concentration of EVs from former NFL player and CTRL CSF: The black line shows the fitting curve. Red line represents the error bar. The y axis is the concentration of particles. The x axis is the size of particle. **(A)** Former NFL player, **(B)** CTRL.

TABLE S1 The sample size of each groups in this study.

TABLE S2 Proteins identified and quantified in TMT-labeled experiment.

TABLE S3 Peptides identified and quantified in TMT-labeled experiment.

## References

[B1] Alosco M. L. Jarnagin J. Tripodis Y. Platt M. Martin B. Chaisson C. E. (2017). Olfactory function and associated clinical correlates in former national football league players. *J. Neurotrauma* 34 772–780. 10.1089/neu.2016.4536 27430424 PMC5314992

[B2] Alosco M. L. Tripodis Y. Fritts N. G. Heslegrave A. Baugh C. M. Conneely S. (2018). Cerebrospinal fluid tau, Aβ, and sTREM2 in former national football league players: modeling the relationship between repetitive head impacts, microglial activation, and neurodegeneration. *Alzheimers Dement* 14 1159–1170. 10.1016/j.jalz.2018.05.004 30049650 PMC6131058

[B3] Asai H. Ikezu S. Tsunoda S. Medalla M. Luebke J. Haydar T. (2015). Depletion of microglia and inhibition of exosome synthesis halt tau propagation. *Nat. Neurosci.* 18 1584–1593. 10.1038/nn.4132 26436904 PMC4694577

[B4] Bieniek K. F. Ross O. A. Cormier K. A. Walton R. L. Soto-Ortolaza A. Johnston A. E. (2015). Chronic traumatic encephalopathy pathology in a neurodegenerative disorders brain bank. *Acta Neuropathol.* 130 877–889. 10.1007/s00401-015-1502-4 26518018 PMC4655127

[B5] Chen Z. Mengel D. Keshavan A. Rissman R. A. Billinton A. Perkinton M. (2019). Learnings about the complexity of extracellular tau aid development of a blood-based screen for Alzheimer’s disease. *Alzheimers Dement* 15 487–496. 10.1016/j.jalz.2018.09.010 30419228 PMC6476313

[B6] Chiasserini D. van Weering J. R. T. Piersma S. R. Pham T. V. Malekzadeh A. Teunissen C. E. (2014). Proteomic analysis of cerebrospinal fluid extracellular vesicles: a comprehensive dataset. *J. Proteom.* 106 191–204. 10.1016/j.jprot.2014.04.028 24769233

[B7] Cicognola C. Brinkmalm G. Wahlgren J. Portelius E. Gobom J. Cullen N. C. (2019). Novel tau fragments in cerebrospinal fluid: relation to tangle pathology and cognitive decline in Alzheimer’s disease. *Acta Neuropathol.* 137 279–296. 10.1007/s00401-018-1948-2 30547227 PMC6514201

[B8] Dayon L. Cominetti O. Wojcik J. Galindo A. N. Oikonomidi A. Henry H. (2019). Proteomes of paired human cerebrospinal fluid and plasma: relation to blood-brain barrier permeability in older adults. *J. Proteome Res.* 18 1162–1174. 10.1021/acs.jproteome.8b00809 30702894

[B9] DeLeo A. M. Ikezu T. (2018). Extracellular vesicle biology in Alzheimer’s disease and related tauopathy. *J. Neuroimmune Pharmacol.* 13 292–308. 10.1007/s11481-017-9768-z 29185187 PMC5972041

[B10] Delpech J.-C. Herron S. Botros M. B. Ikezu T. (2019). Neuroimmune crosstalk through extracellular vesicles in health and disease. *Trends Neurosci.* 42 361–372. 10.1016/j.tins.2019.02.007 30926143 PMC6486849

[B11] Elias J. E. Gygi S. P. (2007). Target-decoy search strategy for increased confidence in large-scale protein identifications by mass spectrometry. *Nat. Methods* 4 207–214. 10.1038/nmeth1019 17327847

[B12] Falcon B. Zivanov J. Zhang W. Murzin A. G. Garringer H. J. Vidal R. (2019). Novel tau filament fold in chronic traumatic encephalopathy encloses hydrophobic molecules. *Nature* 568 420–423. 10.1038/s41586-019-1026-5 30894745 PMC6472968

[B13] Ferreira A. Bigio E. H. (2011). Calpain-mediated tau cleavage: a mechanism leading to neurodegeneration shared by multiple tauopathies. *Mol. Med.* 17 676–685. 10.2119/molmed.2010.00220 21442128 PMC3146621

[B14] Galasko D. R. Shaw L. M. (2017). Alzheimer disease: CSF biomarkers for Alzheimer disease - approaching consensus. *Nat. Rev. Neurol.* 13 131–132. 10.1038/nrneurol.2017.11 28155892 PMC5912691

[B15] Garg S. Timm T. Mandelkow E.-M. Mandelkow E. Wang Y. (2011). Cleavage of Tau by calpain in Alzheimer’s disease: the quest for the toxic 17 kD fragment. *Neurobiol. Aging* 32 1–14. 10.1016/j.neurobiolaging.2010.09.008 20961659

[B16] Gill J. Mustapic M. Diaz-Arrastia R. Lange R. Gulyani S. Diehl T. (2018). Higher exosomal tau, amyloid-beta 42 and IL-10 are associated with mild TBIs and chronic symptoms in military personnel. *Brain Inj.* 32 1277–1284. 10.1080/02699052.2018.1471738 29913077 PMC6129391

[B17] Huttlin E. L. Jedrychowski M. P. Elias J. E. Goswami T. Rad R. Beausoleil S. A. (2010). A tissue-specific atlas of mouse protein phosphorylation and expression. *Cell* 143 1174–1189. 10.1016/j.cell.2010.12.001 21183079 PMC3035969

[B18] Kim D.-K. Lee J. Kim S. R. Choi D.-S. Yoon Y. J. Kim J. H. (2015). EVpedia: a community web portal for extracellular vesicles research. *Bioinformatics* 31 933–939. 10.1093/bioinformatics/btu741 25388151 PMC4375401

[B19] Ko J. Hemphill M. Yang Z. Sewell E. Na Y. J. Sandsmark D. K. (2018). Diagnosis of traumatic brain injury using miRNA signatures in nanomagnetically isolated brain-derived extracellular vesicles. *Lab Chip* 18 3617–3630. 10.1039/c8lc00672e 30357245 PMC6334845

[B20] Lee J. McKinney K. Q. Pavlopoulos A. J. Han M. H. Kim S.-H. Kim H. J. (2016). Exosomal proteome analysis of cerebrospinal fluid detects biosignatures of neuromyelitis optica and multiple sclerosis. *Clin. Chim. Acta* 462 118–126. 10.1016/j.cca.2016.09.001 27609124

[B21] Ling H. Neal J. W. Revesz T. (2017). Evolving concepts of chronic traumatic encephalopathy as a neuropathological entity. *Neuropathol. Appl. Neurobiol.* 43 467–476. 10.1111/nan.12425 28664614

[B22] Manek R. Moghieb A. Yang Z. Kumar D. Kobessiy F. Sarkis G. A. (2018). Protein biomarkers and neuroproteomics characterization of microvesicles/exosomes from human cerebrospinal fluid following traumatic brain injury. *Mol. Neurobiol.* 55 6112–6128. 10.1007/s12035-017-0821-y 29188495 PMC6359938

[B23] Martland H. S. (1928). Punch drunk. *JAMA* 91 1103–1107. 10.1001/jama.1928.02700150029009

[B24] McAlister G. C. Nusinow D. P. Jedrychowski M. P. Wühr M. Huttlin E. L. Erickson B. K. (2014). MultiNotch MS3 enables accurate, sensitive, and multiplexed detection of differential expression across cancer cell line proteomes. *Anal. Chem.* 86 7150–7158. 10.1021/ac502040v 24927332 PMC4215866

[B25] McKee A. C. Cairns N. J. Dickson D. W. Folkerth R. D. Keene C. D. Litvan I. (2016). The first NINDS/NIBIB consensus meeting to define neuropathological criteria for the diagnosis of chronic traumatic encephalopathy. *Acta Neuropathol.* 131 75–86. 10.1007/s00401-015-1515-z 26667418 PMC4698281

[B26] McKee A. C. Stein T. D. Kiernan P. T. Alvarez V. E. (2015). The neuropathology of chronic traumatic encephalopathy. *Brain Pathol.* 25 350–364. 10.1111/bpa.12248 25904048 PMC4526170

[B27] McKee A. C. Stern R. A. Nowinski C. J. Stein T. D. Alvarez V. E. Daneshvar D. H. (2013). The spectrum of disease in chronic traumatic encephalopathy. *Brain* 136 43–64. 10.1093/brain/aws307 23208308 PMC3624697

[B28] Meredith J. E. Sankaranarayanan S. Guss V. Lanzetti A. J. Berisha F. Neely R. J. (2013). Characterization of novel CSF Tau and ptau biomarkers for Alzheimer’s disease. *PLoS One* 8:e76523. 10.1371/journal.pone.0076523 24116116 PMC3792042

[B29] Mez J. Daneshvar D. H. Kiernan P. T. Abdolmohammadi B. Alvarez V. E. Huber B. R. (2017). Clinicopathological evaluation of chronic traumatic encephalopathy in players of American football. *JAMA* 318 360–370. 10.1001/jama.2017.8334 28742910 PMC5807097

[B30] Millspaugh J. A. (1937). Dementia pugilistica. *U.S. Nav. Med. Bull.* 35 297–303.

[B31] Montenigro P. H. Alosco M. L. Martin B. M. Daneshvar D. H. Mez J. Chaisson C. E. (2017). Cumulative head impact exposure predicts later-life depression, apathy, executive dysfunction, and cognitive impairment in former high school and college football players. *J. Neurotrauma* 34 328–340. 10.1089/neu.2016.4413 27029716 PMC5220530

[B32] Moutinho M. Landreth G. E. (2017). Therapeutic potential of nuclear receptor agonists in Alzheimer’s disease. *J. Lipid Res.* 58 1937–1949. 10.1194/jlr.R075556 28264880 PMC5625128

[B33] Nakai W. Yoshida T. Diez D. Miyatake Y. Nishibu T. Imawaka N. (2016). A novel affinity-based method for the isolation of highly purified extracellular vesicles. *Sci. Rep.* 6:33935. 10.1038/srep33935 27659060 PMC5034288

[B34] Nam K. N. Mounier A. Fitz N. F. Wolfe C. Schug J. Lefterov I. (2016). RXR controlled regulatory networks identified in mouse brain counteract deleterious effects of Aβ oligomers. *Sci. Rep.* 6:24048. 10.1038/srep24048 27051978 PMC4823697

[B35] Paulo J. A. Jedrychowski M. P. Chouchani E. T. Kazak L. Gygi S. P. (2018). Multiplexed isobaric tag-based profiling of seven murine tissues following in vivo nicotine treatment using a minimalistic proteomics strategy. *Proteomics* 18:e1700326. 10.1002/pmic.201700326 29660237 PMC5992107

[B36] Perez-Gonzalez R. Gauthier S. A. Kumar A. Levy E. (2012). The exosome secretory pathway transports amyloid precursor protein carboxyl-terminal fragments from the cell into the brain extracellular space. *J. Biol. Chem.* 287 43108–43115. 10.1074/jbc.M112.404467 23129776 PMC3522305

[B37] Perez-Riverol Y. Csordas A. Bai J. Bernal-Llinares M. Hewapathirana S. Kundu D. J. (2019). The PRIDE database and related tools and resources in 2019: improving support for quantification data. *Nucleic Acids Res.* 47 D442–D450. 10.1093/nar/gky1106 30395289 PMC6323896

[B38] Quek C. Hill A. F. (2017). The role of extracellular vesicles in neurodegenerative diseases. *Biochem. Biophys. Res. Commun.* 483 1178–1186. 10.1016/j.bbrc.2016.09.090 27659705

[B39] Stern R. A. Adler C. H. Chen K. Navitsky M. Luo J. Dodick D. W. (2019). Tau Positron-Emission Tomography in Former National Football League Players. *N. Engl. J. Med.* 380 1716–1725. 10.1056/NEJMoa1900757 30969506 PMC6636818

[B40] Stern R. A. Tripodis Y. Baugh C. M. Fritts N. G. Martin B. M. Chaisson C. (2016). Preliminary study of plasma exosomal tau as a potential biomarker for chronic traumatic encephalopathy. *J. Alzheimers Dis.* 51 1099–1109. 10.3233/JAD-151028 26890775 PMC4833534

[B41] Street J. M. Barran P. E. Mackay C. L. Weidt S. Balmforth C. Walsh T. S. (2012). Identification and proteomic profiling of exosomes in human cerebrospinal fluid. *J. Transl. Med.* 10:5. 10.1186/1479-5876-10-5 22221959 PMC3275480

[B42] Tagge C. A. Fisher A. M. Minaeva O. V. Gaudreau-Balderrama A. Moncaster J. A. Zhang X.-L. (2018). Concussion, microvascular injury, and early tauopathy in young athletes after impact head injury and an impact concussion mouse model. *Brain* 141 422–458. 10.1093/brain/awx350 29360998 PMC5837414

[B43] Tatebe H. Kasai T. Ohmichi T. Kishi Y. Kakeya T. Waragai M. (2017). Quantification of plasma phosphorylated tau to use as a biomarker for brain Alzheimer pathology: pilot case-control studies including patients with Alzheimer’s disease and down syndrome. *Mol. Neurodegener.* 12:63. 10.1186/s13024-017-0206-8 28866979 PMC5582385

[B44] Wilhelmus M. M. M. Grunberg S. C. S. Bol J. G. J. M. van Dam A.-M. Hoozemans J. J. M. Rozemuller A. J. M. (2009). Transglutaminases and transglutaminase-catalyzed cross-links colocalize with the pathological lesions in Alzheimer’s disease brain. *Brain Pathol.* 19 612–622. 10.1111/j.1750-3639.2008.00197.x 18673368 PMC8094859

[B45] Zhang Y. Chen K. Sloan S. A. Bennett M. L. Scholze A. R. O’Keeffe S. (2014). An RNA-sequencing transcriptome and splicing database of glia, neurons, and vascular cells of the cerebral cortex. *J. Neurosci.* 34 11929–11947. 10.1523/JNEUROSCI.1860-14.2014 25186741 PMC4152602

[B46] Zhang Z. Song M. Liu X. Kang S. S. Kwon I.-S. Duong D. M. (2014). Cleavage of tau by asparagine endopeptidase mediates the neurofibrillary pathology in Alzheimer’s disease. *Nat. Med.* 20 1254–1262. 10.1038/nm.3700 25326800 PMC4224595

[B47] Zhao X. Kotilinek L. A. Smith B. Hlynialuk C. Zahs K. Ramsden M. (2016). Caspase-2 cleavage of tau reversibly impairs memory. *Nat. Med.* 22 1268–1276. 10.1038/nm.4199 27723722

