# Supplementary material for: Proteomic Profiling of Extracellular Vesicles Isolated From Cerebrospinal Fluid of Former National Football League Players at Risk for Chronic Traumatic Encephalopathy
Source: Front Neurosci. 2019 Oct 9;13:1059. doi: 10.3389/fnins.2019.01059 (PMC6794346; doi:10.3389/fnins.2019.01059)
Supplement: FIGURE S1 — NTA plot of average size and concentration of EVs from former NFL player and CTRL CSF: The black line shows the fitting curve. Red line represents the error bar. The y axis is the concentration of particles. The x axis is the size of particle. (A) Former NFL player, (B) CTRL. [file Image_1.pdf]

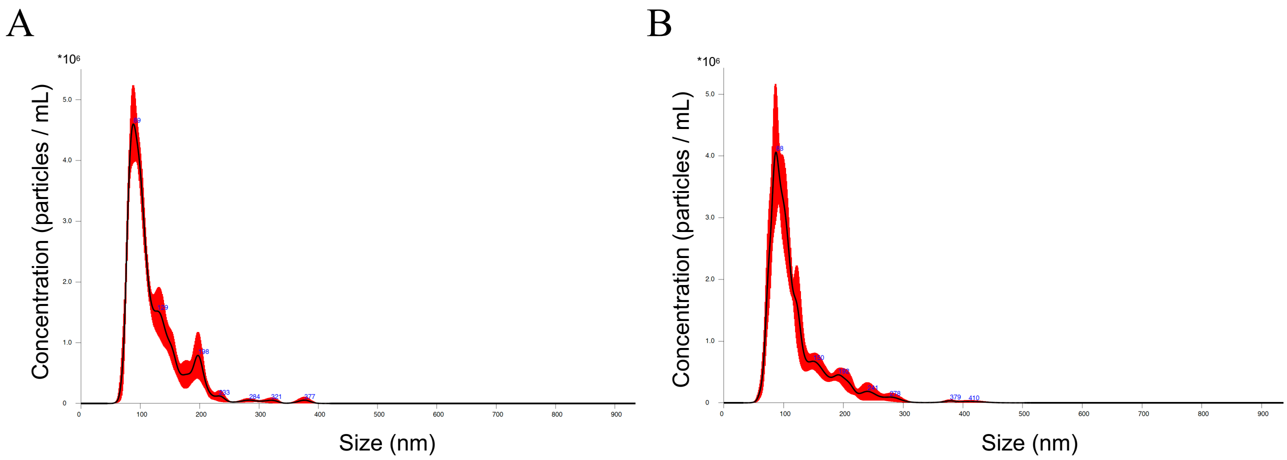

Supplementary Figure S1. NTA plot of average size and concentration of EVs from former NFL player and CTRL CSF: The black line shows the fitting curve. Red line represents the error bar. The y axis is the concentration of particles. The x axis is the size of particle. **(A)** CTRL **(B)** former NFL player
